# Chemopreventive effects of berberine on intestinal tumor development in *Apc*^min/+^ mice

**DOI:** 10.1186/1471-230X-13-163

**Published:** 2013-11-27

**Authors:** Hailong Cao, Shuli Song, Hui Zhang, Yujie Zhang, Rui Qu, Boli Yang, Yang Jing, Tianhui Hu, Fang Yan, Bangmao Wang

**Affiliations:** 1 Department of Gastroenterology and Hepatology, General Hospital, Tianjin Medical University, 154 Anshan Road in Heping District, Tianjin 300052, China; 2 Department of Pathology, General Hospital, Tianjin Medical University, Tianjin 300052, China; 3 Cancer Research Center, Xiamen University Medical College, Xiamen 361005, China; 4 Department of Pediatrics, Division of Gastroenterology, Hepatology and Nutrition, Vanderbilt University Medical Center, Nashville, TN 37232, USA

**Keywords:** Berberine, Intestinal tumor, Proliferation, Apoptosis, Wnt, Epidermal growth factor receptor, *Apc*^min/+^ mouse

## Abstract

**Background:**

Berberine, an isoquinoline alkaloid, has shown inhibitory effects on growth of several tumor cell lines *in vitro.* The aim of this study was to investigate chemopreventive effects of berberine on intestinal tumor development in *Apc*^min/+^ mice.

**Methods:**

Four-week old *Apc*^min/+^ mice were treated with 0.05% or 0.1% berberine in drinking water for twelve weeks. The number and the size of tumors were measured to evaluate intestinal tumor development. Tissue sections were prepared for PCNA and Ki-67 immunostaining to detect cell proliferation, and TUNEL assay and cleaved caspase-3 immunostaining for apoptosis. Western blot analysis and immunostaining were performed to detect the activation of Wnt and epidermal growth factor receptor (EGFR) signaling pathways and COX-2 expression in the intestinal tumor cells. The prostaglandin E_2_ level in the small intestine was detected using ELISA.

**Results:**

Compared with untreated *Apc*^min/+^ mice, the total numbers of tumors in the small intestine and the colon were reduced by 39.6% and 62.5% in 0.05% and 0.1% berberine-treated mice, respectively. The numbers of tumors in proximal, middle, and distal segments of the small intestine in 0.1% berberine-treated mice were significantly reduced by 53.7%, 55.3%, and 76.5% respectively. Berberine treatment also decreased the numbers of all sizes of tumors (>2 mm, 1–2 mm, and <1 mm) in the small intestine. Berberine suppressed tumor cell proliferation and increased apoptosis. Furthermore, berberine decreased the activation levels of Wnt and EGFR signaling pathways, and down-regulated COX-2 expression in intestinal tumor cells and prostaglandin E_2_ production in the small intestine.

**Conclusions:**

Berberine inhibits intestinal tumor development, which is correlated with its activity to suppress tumor cell proliferation and increase apoptosis in *Apc*^min/+^ mice. Down-regulation of Wnt and EGFR signaling pathways and COX-2 expression by berberine may be involved in its anti-tumorigenic effects.

## Background

Berberine, an isoquinoline alkaloid, is present in several plants, such as *Hydrastis canadensis* (goldenseal), *Berberis aquifolium* (Oregon grape), and *Berberis vulgaris* (barberry). Berberine has a wide range of pharmacologic effects, including antimicrobial activities, lipid-lowering efficacy, and regulation of inflammatory responses [1,2]. Recent studies have demonstrated that berberine has antineoplastic effects on several tumor cell lines *in vitro*, including gastrointestinal and liver tumor cells, through inhibiting cancer cell proliferation, inducing caspase-dependent/independent apoptosis, and attenuating reactive oxygen species production [3-7].

However, information regarding berberine’s effects on intestinal tumor development *in vivo* remains limited. The *Apc*^*min/+*^ mouse carries a germline mutation of the mouse adenomatous polyposis coli (*Apc*) gene. The *Apc* gene is a tumor-suppressor gene homologous to the human *Apc*, and mouse carrying this mutation can spontaneously develop multiple tumors in the intestine [8,9]. Since the *Apc* gene mutation plays a significant role in the pathogenesis of familial adenomatous polyposis and sporadic intestinal cancers in patients, *Apc*^min/+^ mouse model is considered as an ideal genetic animal model mimicking human intestinal tumorigenesis. Thus, we used this model for studying chemopreventive effects of berberine on intestinal tumor development.

Several signaling pathways have been reported to promote tumor development in intestinal tumorigenesis, including Wnt and epidermal growth factor receptor (EGFR) [10-12]. *Apc* gene which functions to target β-catenin for degradation has been linked to Wnt signaling pathway. Importantly, it has been shown that *Apc* deficiency is associated with increased EGFR activity in intestinal tumor of *Apc*^min/+^ mice [13]. Moreover, down-regulation of these activated signaling pathways has been reported to effectively inhibit growth of colon cancer cells in humans and tumor formation in *Apc*^min/+^ mice [12,14]. This study was aimed to investigate chemopreventive effects and potential mechanisms of berberine on intestinal tumor development in *Apc*^min/+^ mouse model.

## Methods

### Mice and treatment

*Apc*^min/+^ mice on C57BL/6J background (purchased from Animal Model Institution of Nanjing University, P. R. China) were housed on a 12-h light and 12-h dark cycle. According to dosages in published reports [2,4-6], 0.05% or 0.1% Berberine chloride (Sigma-Aldrich, St. Louis, MO, USA) in drinking water was administered to 4-week old *Apc*^min/+^ mice for 12 weeks. Untreated *Apc*^min/+^ mice were used as the control. Mice were monitored daily for signs of illness. Body weight was recorded weekly. All animal experiments were performed according to the protocol approved by the Institutional Animal Care and Use Committee at Tianjin Medical University, Tianjin, P. R. China.

### Analysis of intestinal tumor development

Analysis of intestinal tumor development was performed as previously described [14-17]. The small intestine and colon were examined using an Olympus SZX7 stereo dissecting microscope. Tumors were categorized as large (>2 mm), medium (1–2 mm), and small (<1 mm). The number of tumors was recorded by the same investigator blinded to the treatment. Swiss-rolled whole small intestine and colon and the liver and the kidney tissues were fixed in 10% formalin for preparing Paraffin-embedded tissue sections. Tissue sections were stained with hematoxylin and eosin and observed under the light microscope to assess tumor stage and toxicity of the liver and the kidney. Tissue sections were also used for immunostaining.

### Immunohistochemistry

To unmask antigens, the small intestinal sections were boiled for 15 min in Antigen Unmasking Solution (Vector laboratories, Inc. Burlingame, CA, USA). Endogenous peroxidase activity was blocked by incubation of sections in 3% H_2_O_2_ for 5 minutes. After blocking non-specific binding using 10% goat serum for 1 h, tissue sections were incubated with primary antibodies, mouse monoclonal anti-proliferating cell nuclear antigen (PCNA, Dako, Glostrup, Denmark), Ki-67 (Novocastra, New Castle, UK), and rabbit polyclonal anti-cleaved caspase-3 (Cell Signaling Technology, Beverly, MA, USA), β-catenin (Santa Cruz Biotechnology, Inc., Santa Cruz, CA, USA), cyclin D1 (Thermo Scientific Inc., Waltham, MA, USA), EGFR and phospho-EGFR (Tyr1068) (p-EGFR, Cell Signaling), phospho-extracellular-signal-regulated kinase (p-ERK, Cell Signaling), phospho-Akt (p-Akt, Ser473) (Cell Signaling), and cyclooxygenase-2 (COX-2, Cell Signaling). Then sections were incubated with peroxidase-labeled anti-mouse IgG or anti-rabbit IgG antibody and developed using DAB. The tissue sections were evaluated under the light microscope by the same investigator blinded to the treatment. At least three tumors in each mouse and five fields at 400X for each tumor were randomly selected for counting the number of positively stained cells. None of the selected fields were overlapped. Data were quantified by calculating percentage of the positive cell in each tumor, then obtaining the average of the percentages of positive cells in each tumor. A modified semiquantitative scoring system was used to calculate COX-2 immunoreactivity scoring, a scale of 0–4 for determining the percentage of positive cells (0, no staining; 1+, <25% of the epithelium stain positive; 2+, 25–50% positive; 3+, 50–75% positive; and 4+, >75% positive), and a scale of 0–3 for strength of intensity of staining (0, no staining; 1+, mild; 2+, moderate; and 3+, intense). The result was presented by the percentage scale X the strength of intensity scale.

### TUNEL assay

Terminal deoxynucleotidyl transferase dUTP nick end labeling (TUNEL) assay (Roche Applied Science, Mannheim, Germany) was used to detect apoptotic cells [14-16]. Apoptotic cells were determined by counting percentage of positive-stained cells in five randomly selected fields in each tumor. At least three tumors in each mouse were randomly selected.

### Preparation of tissue extracts

The tumor tissues from the distal small intestine were solubilized using Cell Lytic™ MT mammalian tissue lysis/extraction reagent (Sigma-Aldrich) and homogenized. The lysates were centrifuged, and the supernatants were retained as total tissue extracts.

Nuclear fractions were prepared using the Nuclear Extraction Kit (Signosis, Inc., Sunnyvale, CA, USA). Briefly, tumor tissues were cut into small pieces, rinsed twice with PBS. Then tissues were solubilized in Buffer I working reagent (10 mmol/L HEPES (pH 7.9), 10 mmol/L KCl, 1.5 mmol/L MgCl_2_, 1 mmol/L EDTA, supplemented with 1 mmol/L DTT and 1 mmol/L protease inhibitor] and homogenized until a single cell suspension was observed microscopically. After centrifugation, pellets were washed and resuspended in Buffer I. After shaking and centrifugation, the nuclear pellets were resuspended in Buffer II working reagent supplemented with 1 mmol/L DTT and 1 mmol/L protease inhibitor. Samples were centrifuged and the supernatant (nuclear extract) was frozen at −80°C.

### Western blot analysis

The protein concentrations were determined using Bicinchoninic acid protein assay (Thermo Scientific Inc.). The total cellular lysates and nuclear fractions were mixed with Laemmli sample buffer for SDS-polyacrylamide gel electrophoresis, and Western blot using primary antibodies against β-catenin, EGFR, p-EGFR and COX-2, and then blotted with horseradish peroxidase conjugated second antibodies. The membranes were visualized using ECL (GE Healthcare, Bucks, UK). Anti-β-actin antibody (Sigma-Aldrich) was used as a total cellular lysate loading control. Anti-histone H3 antibody (Cell signaling) was used as a nuclear protein loading control. The band density was detected using an image processor program (Image J), and was determined by comparing the density of the indicated band to the internal control band of the same mouse.

### ELISA

Normal distal small intestinal mucosal tissues were collected for detecting the prostaglandin E_2_ level (PGE_2_) using ELISA kit (Cayman Chemical Company, Ann Arbor, MI, USA) according to the manufacturer’s instruction, as previously described [9].

### Statistical analysis

Statistical analyses were performed using SPSS (version 13.0; Chicago, IL, USA) for Windows. Means and standard deviation were calculated for continuous variables. The multiplicity of intestinal tumors in each group was compared using One-way *ANOVA* analysis, followed by a *post hoc* multiple comparisons test. Student’s *t*-test was used to compare the percentage of positively stained cells or the fold changes of the ratio for the relative density of bands of berberine-treated group with those of untreated group. The level of statistically significance was set at two-tailed *P* < 0.05.

## Results

### Evaluation of side effects in *Apc*^min/+^ mice treated with berberine

There was no mortality throughout the treatment period in untreated and in 0.05% and 0.1% berberine-treated *Apc*^min/*+*^ mice. We did not find any difference of food and fluid intake and growth rate in berberine-treated mice, compared to untreated *Apc*^min/+^ mice. No histopathologic alterations were found in the liver and the kidney of berberine-treated mice. Thus, no significant side effects by berberine treatment used in this study were found.

### Berberine inhibits intestinal tumor development in *Apc*^min/+^ mice

**Figure 1:**
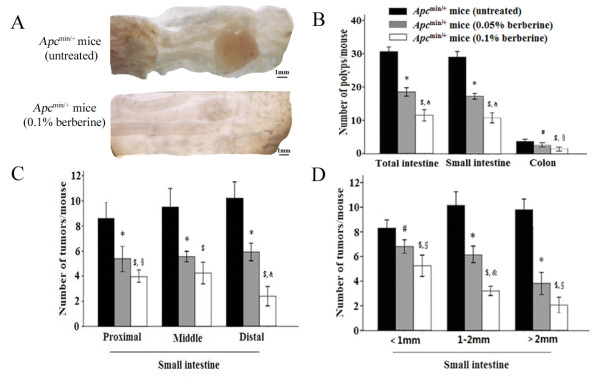
**Berberine inhibits intestinal tumor development in *Apc***^**min/+ **^**mice. (A)***Apc*^min/+^ mice (age, 4 weeks) were treated with berberine, and sacrificed after 12 weeks. The representative gross appearance of the intestinal tumors from untreated and 0.1% berberine-treated *Apc*^min/+^ mice was shown under an Olympus SZX7 stereo dissecting microscope. **(B)** The number of tumors/mouse in the small intestine and colon in 0.05% and 0.1% berberine-treated groups were compared with the untreated *Apc*^min/+^ mice. **(C-D)** Tumor numbers in each section of the small intestine, and size distribution in each group were also listed. Columns, mean from the ten mice in each group; bars, standard deviation. *, *P* < 0.01 and ^#^, *P* < 0.05, 0.05% berberine-treated *vs* untreated *Apc*^min/+^ mice; ^$^, *P* < 0.01 and ^※^, *P* < 0.05, 0.1% berberine-treated *vs* untreated *Apc*^min/+^ mice; &, *P* < 0.01 and ^§^, *P* < 0.05, 0.1% berberine *vs* 0.05% berberine-treated *Apc*^min/+^ mice.

We first evaluated tumor development in 16-week old *Apc*^*min/+*^ mice with or without 12-week berberine treatment. Berberine significantly reduced the multiplicity of intestinal tumor in *Apc*^min/+^ mice in a dose-dependent manner (Table 1, Figure 1). Compared with the total number of tumor in the small intestine and the colon in untreated *Apc*^min/+^ mice (30.63 ± 1.69), 0.05% and 0.1% berberine treatment significantly decreased the number of tumor by 39.6% (18.50 ± 1.51, *P* < 0.01), and 62.5% (11.50 ± 2.05, *P* < 0.01, Figure 1B), respectively. Berberine reduced 62.0% and 31.2% of the number of colon tumor in 0.05% and 0.1% treatment groups (Figure 1B). In addition, tumor numbers in proximal, middle, and distal portions of the small intestine in 0.1% berberine-treated group were reduced by 53.7%, 55.3%, and 76.5%, respectively, while those in 0.05% group were reduced by 37.4%, 41.5%, and 42.0%, respectively. The numbers of all sizes of tumors (>2 mm, 1–2 mm, and <1 mm) in the small intestine were significantly reduced (Figure 1D). Adenomas with or without low-grade dysplasia were found in the small intestine and colon in untreated and berberine-treated *Apc*^*min/+*^ mice, and we did not find the difference regarding tumor stage in these two groups (data not shown).

**Table 1:** **Effects of berberine on intestinal tumor multiplicity in *Apc***^**min/+ **^**mice (**$\bar{x}\pm S$**, *n*** **= 10)**

	**Untreated mice**	**0.05% berberine**	**0.1% berberine**
**Total number**	30.63 ± 1.69	18.50 ± 1.51*	11.50 ± 2.05^$, &^
**Small intestinal**	28.00 ± 2.00	16.69 ± 1.03*	10.50 ± 1.77^$, &^
Proximal	8.38 ± 1.51	5.25 ± 1.16*	3.88 ± 0.64^$, §^
Middle	9.50 ± 1.77	5.56 ± 0.50*	4.25 ± 1.04^$^
Distal	10.13 ± 1.55	5.88 ± 0.83*	2.38 ± 0.92^$, &^
<1 mm	8.13 ± 0.83	6.81 ± 0.65^#^	5.25 ± 1.04^$, §^
1-2 mm	10.13 ± 1.36	6.19 ± 0.84*	3.25 ± 0.46^$, &^
>2 mm	9.75 ± 2.05	3.69 ± 1.03*	2.00 ± 0.76^$, §^
**Colon**	2.63 ± 0.74	1.81 ± 0.65^#^	1.00 ± 0.46^$, §^

*, *P* < 0.01 and ^#^, *P* < 0.05, 0.05% berberine-treated *vs* untreated *Apc*^min/+^ mice; ^$^, *P* < 0.01 and ^※^, *P* < 0.05, 0.1% berberine-treated *vs* untreated *Apc*^min/+^ mice; ^&^, *P* < 0.01 and ^§^, *P* < 0.05, 0.1% berberine *vs* 0.05% berberine-treated *Apc*^min/+^ mice.

### Berberine suppresses cell proliferation and increases apoptosis in intestinal tumors

**Figure 2:**
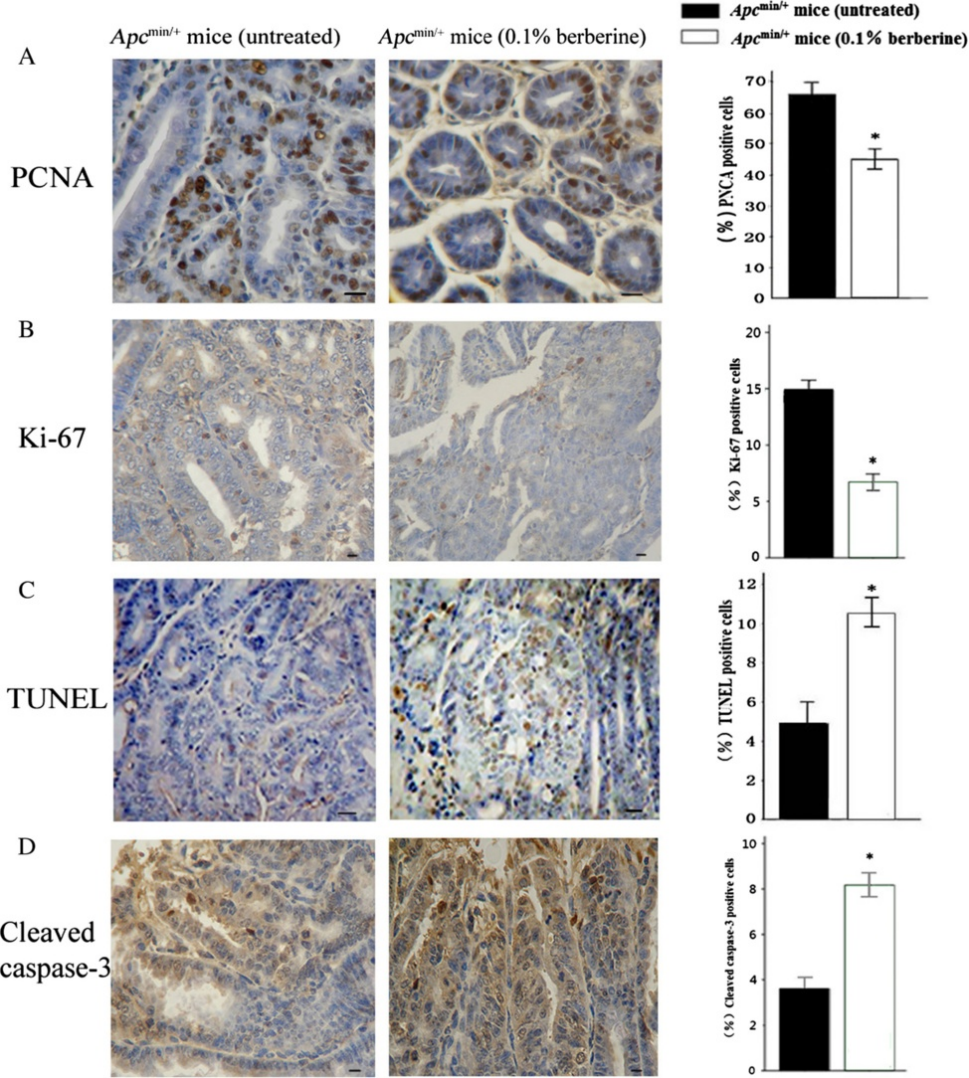
**Berberine reduces proliferation and induced apoptosis in intestinal tumors of *Apc***^**min/+ **^**mice. (A-B)** Distal small intestinal sections from untreated and 0.1% berberine-treated mice were stained with PCNA and Ki-67. **(C-D)** Small intestinal segments from untreated and 0.1% berberine-treated groups were processed for TUNEL and cleaved caspase-3 staining (400×). Data were quantified as mean percentage of positive cells at five randomly selected fields in each sample. Scale bars, 50 μm. Columns, means from at least six mice in each group; bars, standard deviation. *, *P* < 0.01, 0.1% berberine-treated *vs* untreated *Apc*^min/+^ mice.

To further evaluate mechanisms of berberine’s effects on intestinal tumor development in *Apc*^*min/+*^ mice, we assessed tumor cell proliferation and apoptosis (Figure 2). We focused on comparison between samples from untreated *Apc*^min/+^ mice and 0.1% berberine-treated group, which showed the higher degree inhibition of intestinal tumor development. Immunostaining showed that berberine significantly decreased PCNA (44.60 ± 2.88 *vs* 65.80 ± 3.27, by 32%) and Ki-67 (6.73 ± 2.16 *vs* 14.89 ± 2.75, by 55%) positive cells (Figure 2A-B), suggesting that berberine inhibits tumor cell proliferation in *Apc*^*min/+*^ mice.

The apoptotic cells in tumors detected by TUNEL staining were significantly increased by 2.14 fold in berberine-treated mice compared with that in the untreated mice (10.52 ± 0.61 *vs* 4.92 ± 0.86, *P* < 0.01, Figure 2C). Moreover, berberine treatment increased caspase-3 activation detected by cleavage caspase-3 immunostaining of intestinal tissues (8.18 ± 1.45 *vs* 3.60 ± 1.34, *P* < 0.01, Figure 2D). Thus, these results suggest that inhibition of proliferation and promotion of apoptosis by berberine may play a role in the prevention of tumor development in *Apc*^*min/+*^ mice.

### Berberine regulates signaling pathways in intestinal tumor of *Apc*^min/+^ mice

**Figure 3:**
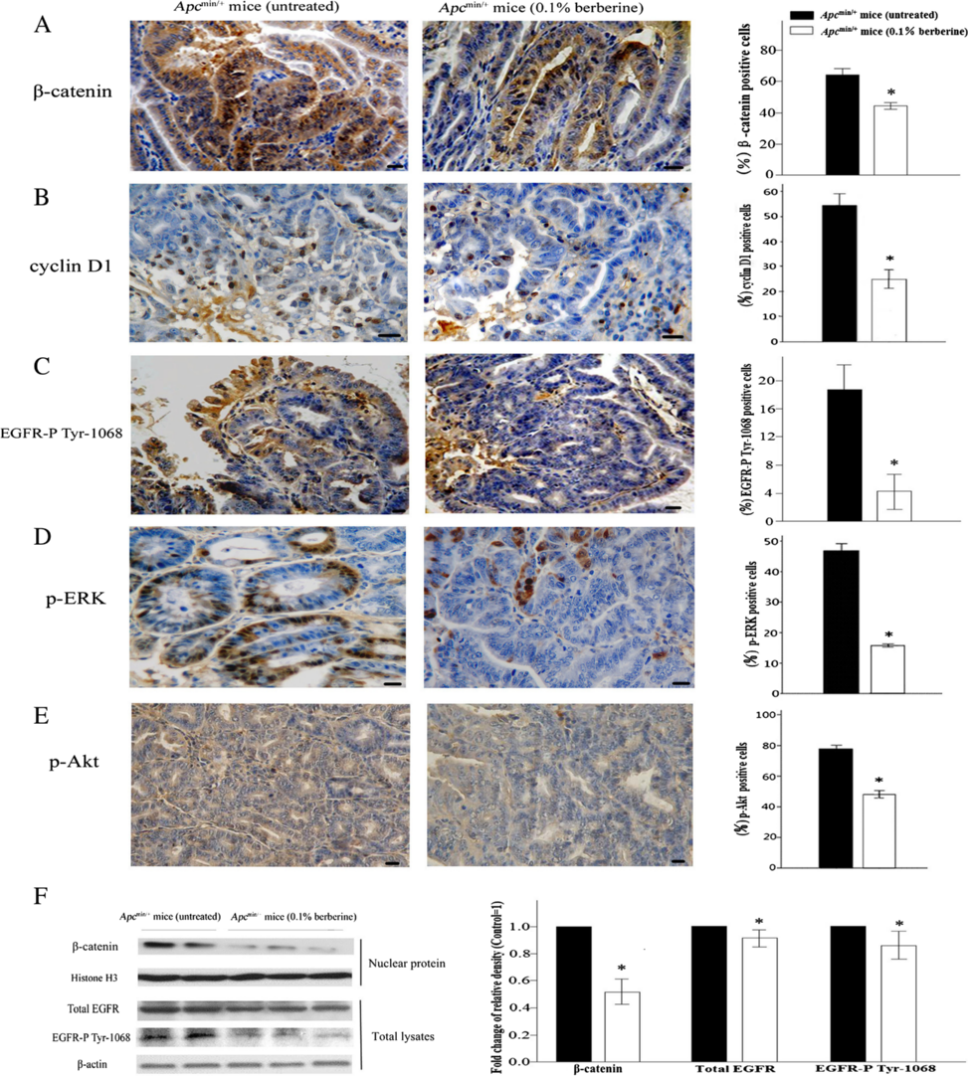
**Berberine treatment regulates signaling pathways in intestinal tumors of *Apc***^**min/+ **^**mice. (A-B)** Immunohistochemical analyses of tumors using antibodies against β-catenin and cyclinD1 from the distal small intestine of untreated and 0.1% berberine-treated mice were shown (400×). Scale bars, 50 μm. Data were quantified as mean percentage of positive cells for β-catenin in cytoplasm and/or nuclear and cyclinD1 at five randomly selected fields. **(C-E)** p-EGFR, p-ERK and p-Akt were also analyzed by immunohistochemical staining. Data were quantified as mean percentage of positive cells. **(F)** Nuclear protein extracts were prepared from tumors, and analyzed by Western blot analysis using anti-β-catenin antibody. Anti-histone H3 antibody was used as a nuclear protein loading control. Protein lysates were prepared and analyzed by Western blot analysis using anti-total EGFR and p-EGFR antibodies, and anti-β-actin antibody was used as a protein loading control. The protein band ratio was calculated by comparing the relative density of the protein band for β-catenin, total EGFR, and p-EGFR to that of internal control band from the same mouse. The average ratio in control was set as 100%, the fold changes of the ratio in treated mice were shown. Columns, means from at least six mice in each group; bars, standard deviation. *, *P* < 0.01, 0.1% berberine-treated *vs* untreated *Apc*^min/+^ mice.

We further studied the effects of berberine on signaling pathways involved in tumor development. It has been reported that cytoplasmic accumulation and nuclear translocation of β-catenin was found in *Apc*^min/+^ mice, which indicated the aberrant Wnt signaling. Immunostaining showed that berberine treatment decreased the percentage of positive cells of β-catenin in cytoplasm and/or nuclear of intestinal tumor in *Apc*^min/+^ mice (43.20 ± 1.63 *vs* 63.00 ± 3.08, *P* < 0.01, Figure 3A). Cell cycle arrest is associated with the expression of cell cycle regulators. Decreased cyclin D1, one of the key downstream molecules of Wnt signaling, promotes G1 arrest. Berberine reduced expression of cyclin D1 by 54% (24.80 ± 3.11 *vs* 54.40 ± 3.78, *P* < 0.01, Figure 3B). Furthermore, phosphorylation of EGFR, ERK, and Akt in intestinal tumors was suppressed by berberine treatment. The average percentages of p-EGFR-positive cells in berberine and untreated groups were 4.05 ± 2.74 *vs* 18.80 ± 3.74 (*P* < 0.01, Figure 3C), p-ERK-positive cells are 16.55 ± 1.54 *vs* 46.88 ± 7.25 (*P* < 0.01, Figure 3D), and p-Akt stained cells were 42.13 ± 7.76 *vs* 77.60 ± 8.36 (*P* < 0.01, Figure 3E), respectively.

Moreover, the protein band ratio was calculated by comparing the relative density of the protein band on Western blots for β-catenin, total EGFR, and p-EGFR to that of internal control band from the same mouse. The average ratio in control was set as 100%, the fold changes of the ratio in treated mice were shown. The levels of these proteins were reduced in tumors of berberine-treated *Apc*^*min/+*^ mice compared with that of untreated group (*P* < 0.01, Figure 3F).

### Berberine down-regulates COX-2 expression and PGE_2_ production

**Figure 4:**
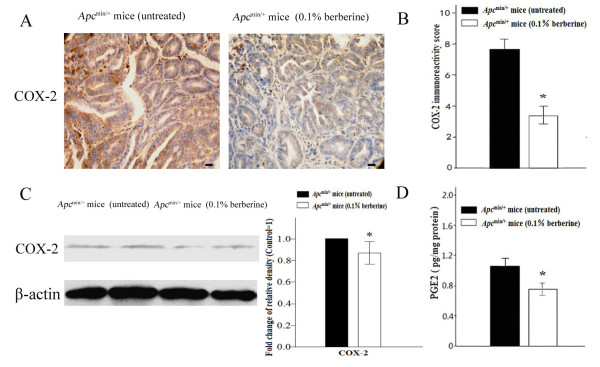
**Berberine down-regulates COX-2 expression and PGE**_**2 **_**production. (A-B)** Tumors of the distal small intestine from untreated and 0.1% berberine-treated groups were immunostained using COX-2 antibody. Immunoreactivity scoring using a modified semiquantitative scoring system was shown (400×). Scale bars, 50 μm. **(C)** Protein lysates were prepared from tumors and analyzed by Western blot analysis. Anti-β-actin antibody was used as a protein loading control. The protein band ratio was calculated by comparing the relative density of the protein band on Western blots for COX-2 to that of internal control band from the same mouse. The average ratio in control was set as 100%, the fold change of the ratio in treated mice was shown. **(D)** PGE_2_ levels of normal mucosa from untreated and 0.1% berberine-treated groups were detected by ELISA. Columns, means from at least six mice in each group for immunohistochemistry and Western blot analysis, and ten mice in each group for ELISA assay; bars, standard deviation. *, *P* < 0.01, 0.1% berberine-treated *vs* untreated *Apc*^min/+^ mice.

Over expression of COX-2 and increased PGE_2_ production were reported to be associated with chronic inflammation and cell proliferation. Berberine significantly down-regulated COX-2 immunoreactivity in small intestinal tumors of *Apc*^min/+^ mice (3.38 ± 0.51 *vs* 7.60 ± 0.57, *P* < 0.01, Figure 4A-B). The expression level of COX-2 was significantly decreased in tumors of berberine-treated *Apc*^*min/+*^ mice, compared with that of untreated group (*P* < 0.01, Figure 4C). Berberine also decreased PGE_2_ production, a downstream product of COX-2, in normal mucosa (0.76 ± 0.08 *vs* 1.06 ± 0.10, *P* < 0.01; Figure 4D). The results suggest that the inhibition of COX-2 and PGE_2_ production by berberine may play a role in chemoprevention of intestinal tumorigenesis.

## Discussion

Evaluation of ancient natural products that have been widely used for specific diseases, including berberine, has attracted attention to explore their broad clinical applications. Our studies suggest that berberine has the potential to prevent the growth and recurrence of intestinal precancerous lesions. Although aspirin and some specific COX-2 inhibitors, such as celecoxib, have shown chemopreventive effects on colonic tumor development in animal model and some patients [9,18-21], the potential gastrointestinal and cardiovascular side effects in patients limit their long-term use [22,23]. Berberine has recently emerged as a potential chemopreventive agent due to its antineoplastic effects [3-7]. The *in vitro* activities of berberine may not represent the *in vivo* effects. Therefore, it is important to investigate the efficacy of berberine *in vivo*. The present study provides information regarding effects of berberine on intestinal tumor development using *Apc*^min/+^ mice model. Berberine was well tolerated, and significantly reduced the multiplicity of intestinal tumor in a dose-dependent manner. Berberine reduced cell proliferation and induced apoptosis, which are associated with inhibition of Wnt and EGFR signaling pathways in intestinal tumor of *Apc*^min/+^ mice. Our data also showed that berberine down-regulated COX-2 activity. These results, together with the recent study of prevention of azoxymethane-induced colon cancer by berberine in rats [24], support the chemopreventive effects of berberine on intestinal tumor.

Excessive proliferation and insufficient apoptosis are related to intestinal tumor development and progression [14,25-27]. We found that berberine inhibited cell proliferation and induced apoptosis in tumor in *Apc*^min/+^ mice by regulating signaling pathways involved in proliferation and apoptosis. Antiproliferative and proapoptotic effects play roles in inhibition of intestinal tumor formation and growth by berberine. One of the most important observations in the present study is that larger size tumors (>2 mm in the small intestine) were significantly decreased by berberine, suggesting that berberine exerts strong activity to limit progression of polyps.

Dysregulation of Wnt signaling pathway has been reported to play a major role in intestinal tumorigenesis in humans and *Apc*^min/+^ mice [10,14,28]. Truncated APC protein encoded by mutated gene causes cytoplasmic accumulation and nuclear translocation of β-catenin to transactivate T cell factor/lymphoid enhancer factor in the nucleus, leading to increasing transcription of many oncogenic genes including cyclin D1 [10]. Our data showed that the increased expressions of β-catenin in cytoplasm and nuclear in *Apc*^min/+^ mice were significantly decreased by berberine, which supports berberine’s role in antiproliferative mechanisms. These results are in consistence with the findings from the *in vitro* study showing that berberine inhibits the proliferation of colon cancer cells by inactivating Wnt/β-catenin signaling [29]. Further studies are required to characterize the exact mechanisms underlying berberine’s inhibitory effects on Wnt signaling, such as whether berberine inhibits β-catenin translocation into the nucleus or enhances β-catenin degradation.

EGFR signaling pathway activation is another key process in the development and progression of many tumors, including intestinal cancer [30]. Interestingly, Roberts *et al*. reported that *Apc*^min/+^ mice carrying an EGFR mutation with a marked reduction in EGFR activity had a 90% reduction in intestinal tumor compared with *Apc*^min/+^ mice expressing normal EGFR [31]. Recently, increasing evidence indicates that both Wnt and EGFR signaling pathways mediate β-catenin activation [32]. Aberrant Wnt pathway triggers pro-survival/anti-apoptotic signaling cascades activation such as phosphatidylinositol 3-kinase/Akt pathway [33,34]. We analyzed the activation of EGFR signaling pathway in intestinal tumors of *Apc*^min/+^ mice. Importantly, berberine treatment could significantly suppress EGFR activation and its downstream targets ERK and Akt. Therefore, we hypothesize that berberine inhibits intestinal tumor development in *Apc*^min/+^ mice through concomitant suppression of EGFR pathway, leading to decreasing tumor cell proliferation and increasing apoptosis.

Over expression of COX-2 and increased PGE_2_ production were reported to be associated with chronic inflammation and endothelial cell proliferation by releasing various angiogenic factors [35,36], and boosting PGE_2_ production was also shown to be related to the polyps expansion [37]. Thus, the inhibition of COX-2 and PGE_2_ production by berberine may also play a role in chemoprevention of intestinal tumorigenesis.

## Conclusions

The present study shows that berberine treatment significantly suppresses spontaneous intestinal tumor development in *Apc*^min/+^ mice, inhibits tumor cell proliferation, and induces apoptosis, which are associated with berberine’s activity to inhibit signaling pathways involved in tumor development. Thus, berberine may be a relatively nontoxic and low-cost agent to prevent intestinal tumor development.

### Ethics approval

This study was conducted with the approval of the Institutional Animal Care and Use Committee at Tianjin Medical University, Tianjin, P. R. China.

## Competing interests

The authors declare that they have no competing interests.

## Authors’ contributions

HC, SS, HZ, YZ, RQ, BY, and YJ carried out the experiments. HC, SS, TH, FY and BW participated in the design of the study and coordination, and drafted the manuscript. All authors read and approved the final manuscript.

## Pre-publication history

The pre-publication history for this paper can be accessed here:

http://www.biomedcentral.com/1471-230X/13/163/prepub
